# Social deprivation in maternal mouthbrooders *Tropheus* sp. “Caramba” (Teleostei: Cichlidae) decreases the success rate of reproduction and survival rate of fish fry

**DOI:** 10.1038/s41598-023-35467-z

**Published:** 2023-05-22

**Authors:** Jindřich Novák, Daniel Frynta, Daniela Nováková, Jiří Patoka

**Affiliations:** 1 Department of Zoology and Fisheries, Faculty of Agrobiology, Food and Natural Resources, Czech University of Life Sciences Prague, Kamýcká 129, 16500 Prague-Suchdol, Czech Republic; 2 Department of Zoology, Faculty of Science, Charles University, Viničná 7, 12844 Prague, Czech Republic; 3 Department of Physiology, Faculty of Science, Charles University, Viničná 7, 12844 Prague, Czech Republic; 4 First Faculty of Medicine, Charles University, Kateřinská 32, 12108 Prague, Czech Republic

**Keywords:** Behavioural methods, Developmental biology, Animal behaviour, Reproductive signs and symptoms

## Abstract

Early offspring separation from mothers causes social deprivation. Mouthbrooding, when eggs and fry are incubated in the buccal cavity of the parent, is one of the reproductive strategies in fish. The mother is the incubating parent in African lake cichlids from the genus *Tropheus*. Many of these are produced in captivity and some producers use artificial incubators in which eggs are incubated separately from the mother. We hypothesized that this practice may dramatically modify the reproduction rate of fish individuals produced by the method of artificial incubation. The long-term experiment focused on *Tropheus* sp. “Caramba” had been carried out for 10 years when maternally incubated and separated individuals were compared. We found a negative effect of artificial egg and offspring incubation out of the mother’s buccal cavity. The deprived females laid the same number of eggs as maternally incubated females, but most eggs were lost during the incubation. Moreover, the reproduction frequency was significantly lower in deprived females in comparison with those maternally incubated. This study should be perceived as preliminary. For this reason and with respect to welfare principles, we strongly recommend similarly designed experiments focused on other potentially sensitive fish mouthbrooders. Once the syndrome would be confirmed, we recommend avoiding artificial incubation of mouthbrooding fish in general.

## Introduction

Early separation from mothers causes social deprivation in offspring^1^. In such deprived individuals, the normal behavioural patterns are modified or interrupted due to lack of adequate opportunity for social experience, and related psychosocial stress affects further individual development leading to irreversible behavioural alterations later in its life. These phenomena were noticed in various animal taxa including mostly vertebrates and certain invertebrate cases and are perceived to be a paradigm^2–6^. Typical symptoms are violent behaviour, disturbed social communication and performance in social interactions, reduced ability to learn socially, altered reproductive behaviour, and higher sensitivity to stress^7^. Moreover, early social deprivation had negative life-long effects on the neurogenomic profile of the affected individuals^8^.

Nevertheless, the mechanisms of how social experiences influence behaviour and brain development of affected individuals have not yet been fully elucidated. It is caused by the lack of full control over social experiences in species with developed parental care, where social isolation and isolation from the parent(s) can disrupt regular individual development. Moreover, social behaviour in most animals is complex of actions triggered by various interacting stimuli that are difficult to isolate and fully understand^9,10^. Hence, it is difficult to distinguish the resulting specific social deficits from general disorders^11^. Inasmuch as especially commercially reared animals are usually separated from their mothers/parents before reaching the age at which they would naturally become free-living^1^, experiments focused on social deprivation in these species are valuable. Since ornamental aquaculture is a very popular hobby with many enthusiasts worldwide^12,13^, the producers are motivated to culture or collect in the fields the demanded species in huge quantities^14–16^. Mouthbrooding cichlids belong to the very popular and most traded ornamental fish taxa and the vast majority of them are produced in captivity^12^.

Mouthbrooding, also known as oral or buccal incubation is one of the parental care strategies to increase offspring survival rate^17^. One parent or both parents incubate a clutch of eggs and possibly also newly hatched embryos in a ventral bulging of the hyoid region until the complete or almost complete adsorption of the yolk sac. The incubating parent can adjust the incubation duration as a response to environmental cues such as the perceived risk of predation^18^. Mouthbrooding is not an uncommon parental care strategy in both freshwater and marine fishes and can be found across various fish taxa. A paternal model is the predominant type and is known for most groups^19^ while maternal and biparental mouthbrooding occur typically in cichlids^20^. Certain stressors such as anthropogenic noise during the mouthbrooding incubation can be negatively reflected in the survival rate of affected offspring^21^.

Both commercial and hobby producers of mouthbrooding fish species also use upwelling incubators (so-called artificial mouths, egg tumblers or clutch-removal method) for fish eggs in the eye spot stage as an appropriate method of multiplying^22–24^. The eggs in the incubator are agitated with a low-head airlift pump during the incubation period^25^. This method is recommended to improve control options and increase the efficiency of fry rearing by producers.

On the other hand, several studies show the negative impacts of artificial incubation on psychosocial development of incubated both invertebrate and vertebrate animals which is contrary to welfare principles and can produce emotionally and psychically disturbed individuals^2,26^. In fish, it was found that the adult social behaviour and neurophysiological development were persistently modified by early life social experience^8,11,27^. Imprinting is a very important sensitive period in fish development^28^ and in several mouthbrooders such as *Oreochromis* spp., it was found that early separation of eggs from the mother caused significant negative impact on filial social bond formation of fry later in its life^29^. The importance of qualitative study of such social deprivation from separation from mother or parents in fish is obvious.

Since social deprivation may negatively affect ornamental aquaculture in general, and welfare of kept mouthbrooders in particular, we decided to survey this syndrome and its possible consequences in the popular cichlids from the genus *Tropheus* native to African Lake Tanganyika^17^, with a special focus on reproduction and survival rate of fish fry. There is no previous record of social deprivation of offspring in these mouthbrooder cichlids. Species of this genus are abundant in rocky habitats in the upper littoral zone where they feed on algae and hide from predators, while sandy or muddy bottom and shores are strictly avoided^30^. From ecological and genetic studies, it follows, that *Tropheus* cichlids have site fidelity and are not able to cross greater distances and unsuitable habitats^31^. These fishes are among the popular ornamental fish species on the market^12,32^. *Tropheus* mouthbrooders form a society with well-expressed linear hierarchy from alpha to omega role of each individual in the tank^33,34^. From the ornamental aquaculture perspective, these mouthbrooders belong to the so-called problematic fish species and, in general, *Tropheus* individuals suffer greatly from inconsiderate handling and frequently die as a consequence of stress^35^. The experiment was designed as a long-term study with a special focus on the effectiveness of mouthbrooding in females of *Tropheus* sp. “Caramba” which were bred under different conditions: maternal incubation (N-females) vs. incubation in an incubator without a mother (D-females).

## Results

Table 1 shows the results of artificial incubation separate from the mother (52 clutches from different N-females and 30 clutches from different D-females). The absolute individual fecundity of N and D-females was almost the same. The mean number of eggs per spawning was 8.07 and 8.13, respectively. The effect of deprivation (N versus D) was not significant (analysis of deviance: null = 80.229, residual = 80.222, resid. df = 80, P = 0.931). Unlike in natural incubation, offspring of both N and D-mothers exhibited high survival rates (78.8% and 94.7%). The higher survival of offspring produced by D-females was significant (glm: analysis of deviance: null = 322.22, residual = 287.97, resid. df = 80, P = 0.0020; the model: intercept_N_ = 1.313, SE = 0.226, t = 5.82, P < 0.0001, coefficient_D_ = 1.564, SE = 0.584, t = 2.68, P = 0.0090).

**Table 1:** Incubation of eggs of *Tropheus* sp. “Caramba” in artificial incubator: Group type (N = “normal” = maternally incubated; D = “deprived” = incubated separately from mother in artificial incubator); numbers of eggs laid: median, range and standard deviation (SD); numbers of offspring per spawning successfully reared: median, range and standard deviation (SD).

Group type	Clutches	Eggs laid			Reared offspring per spawning		
		Median	Range	SD	Median	Range	SD
N	52	8	4–15	3.0	7	0–15	3.7
D	30	8	4–14	2.9	8	2–11	3.1

**Figure 1:**
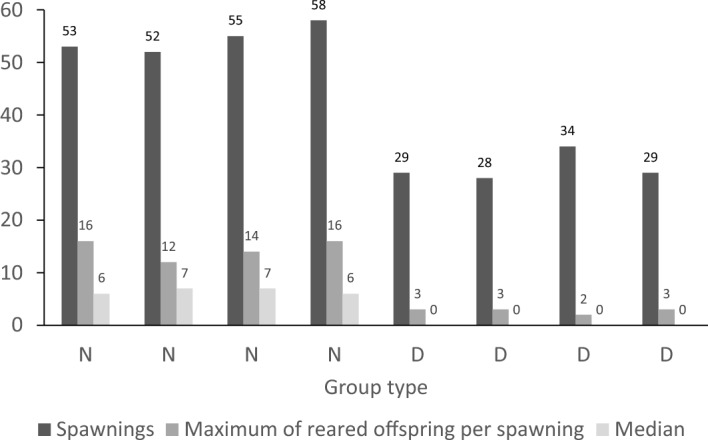
Comparison of reproduction rate of maternally incubated and socially deprived females of *Tropheus* sp. “Caramba”: Group type (N = “normal” = maternally incubated; D = “deprived” = incubated separately from mother in artificial incubator); Spawnings = total number and median of spawnings of all females in each group within the period of 2 years; maximum numbers of offspring per spawning successfully reared by mothers.

In total, four N and four D-groups of *Tropheus* sp. “Caramba” females completed the whole experimental period and were subsequently analysed. Within the experimental period, both N and D-females increased their size by about 1–2 cm. In each group consisting of 15 N-females we recorded 52–58 spawnings during the period of 2 years, but in corresponding groups of D-females only 29–34 spawnings (Fig. 1, Table S1). Geeglm (Generalized Estimating Equation Generalized Linear Model) confirmed that the D-females spawned less often than N-females (anova: D vs N: df = 1, χ^2^ = 181, P < 0.0001; the model: intercept_N_ = 1.290, S.E. = 0.021, Wald = 3767, P < 0.0001; coefficient_D_ = − 0.597, S.E. = 0.044, Wald = 181, P < 0.0001).

All of 60 N-females and 38 of 60 D-females reproduced (218 N and 120 D spawnings) within the experimental period. The N-females produced up to 16 eggs in one spawning (about eight on average) and released 6–7 hatchlings on average after the entire incubation. The D-females produced similar number of eggs in one spawning (up to 14, on average eight to nine) but released a maximum of three, but mostly no juveniles (Fig. 1, Table S1). Thus, during the natural incubation, N-females eat or lose only two embryos on average, while D-females lose up to nine embryos. Geeglm revealed that the negative effect of mother deprivation on the number of successfully reared embryos per spawning was highly significant (anova: df = 1, χ^2^ = 387.7, P < 0.0001). D-mothers reared less embryos per spawning (coefficient_D_ = − 1.935, SE = 0.107, W = 327.4, P < 0.0001) than N-mothers (intercept_N_ = 1.968, SE = 0.040, W = 2467.9, P < 0.0001). 74.4% of variation in the number of successfully reared embryos was attributable to the effect of deprivation (21.4% to residual variation among clutches of the same mother).

## Discussion

The negative effect of artificial egg and offspring incubation outside the mother’s buccal cavity was found in mouth-brooding cichlids *Tropheus* sp. “Caramba”. Our finding is the first document with quantitative data that showed the deprivation of social separation in parental care fish. We believe that our findings will bring a new insight to the production and multiplying of ornamental mouthbrooding fish species in captivity with possible overlap to the management practice of their culture and breeding. The offspring were obviously socially deprived by this practice and their behaviour was significantly disturbed, causing alteration in the maternal care of eggs and offspring. The D-females laid the same portion of eggs as N-females but D-females lose a majority of their clutch during incubation. Moreover, the reproduction frequency was remarkably lower in deprived females in comparison with those maternally incubated. On the other hand, in the case of artificial incubation, the numbers of successfully hatched fish were similar despite the origin of N or D-mothers and one can conclude that eggs are viable in both groups. Therefore, the numbers of offspring successfully hatched from eggs laid by D-mothers are higher when incubated in an incubator in comparison with those incubated by a mother. Nevertheless, individuals incubated separately from the mother are deprived and it is probable that they will have negatively affected reproduction later in life. It is evident that N-mothers incubating their eggs in the buccal cavity is the most successful mode and their offspring will behave “normally”, i.e. their reproduction will not be affected.

Even if it is clear that in comparison with maternally incubated females, the deprived females of *Tropheus* sp. “Caramba” failed to multiply, the direct cause is unknown. The mechanical process of the deprivation might be more important than revealing just direct physical factors. The process in the individual development can be related to emotional or mental aspects, which will be comparable to the cases of mammals such as primates^36^: the cichlid infants receiving maternal care will feel safety, easiness or comfortableness in the close relationship with the caretaker. Moreover, some fish feel comfortableness when receiving tactile massage from other fish, reducing stress hormone^37^. The relationship between mother and offspring within the buccal incubation covers various aspects including a complex of tactile, olfactory and visual communication which can decrease stress of embryos and hatchlings^35,38^. Also, mutual chemical perception among the embryos and mother cannot be excluded.

It is worth mentioning that little is known about post pharyngeal care duration (the time between first release of fry and last guarding of young) of *Tropheus* cichlids. These taxa are semelcavus^39^ in the wild, i.e. once the fry are released they are never taken back into the mother’s buccal cavity^40^. However, the same species can show the iterocavus mouthbrooding mode (released fry re-enter parent’s mouth to escape danger or to overnight)^39^ when kept in a limited space, e.g. in aquaria; after the fry are released, young fish remain close to their mother and will re-enter her buccal cavity when endangered, over an extended period of an additional 3–5 days^40,41^. Thus, maternal care in the model species is obviously more prominent and prolonged in aquaria. For this reason, in comparison with the semelcavus wild fish, the social deprivation symptoms are more intensively manifested affecting the success rate of reproduction and survival of offspring.

Even if our knowledge about potential behavioural disruption in other mouthbrooders remains limited, one can expect similar patterns as were found in our study. Thus, not only in cichlids, mouth-brooding had reported from at least other eight families of teleosts^20,42–44^. Certain brood parasites such as cuckoo catfishes of the genus *Synodontis* (family Mochokidae) are evolutionary mouthbrooders and their eggs and offspring can be affected by social deprivation even if incubated by females of different fish taxa^45^. Indeed, gill chamber brooder cavefishes (family Amblyopsidae) can also be considered from this perspective^46^. Further research focused on this issue is recommended to ascertain the importance of mouthbrooding care in mentioned taxa.

Therefore, the method of artificial incubation of eggs and offspring of mouthbrooders without the mother can cause huge production of socially deprived and thus abnormally behaving fish individuals, at least in the surveyed taxon. The egg or filial cannibalism was reported in mouthbrooding cichlids if the number of eggs in the mouth falls below a critical value of approximately 20% of the total number of eggs spawned or as a consequence of experimentally increased brood size and changed brood composition^47^. Since the deprived females lose or eat most of their own eggs, the producers of ornamental fish frequently separate the eggs from mothers and incubate them in artificial incubators, aiming for the production of higher numbers of hatchlings, which can be effective as was confirmed by our experiment. Moreover, the parent fish can instantly perform the next spawning behaviour because they do not have to do mouthbrooding. This method is thus recommended as beneficial by some authors^22^. It is obvious that they are caught in a vicious cycle—when more deprived fish are produced, the demand for artificial incubation individuals increases, and this will lead to higher production of deprived fish. On the other hand, N-females are able to maternally incubate eggs with similar success rate as in an artificial incubator. Even if survival rate of maternally incubated and not-deprived offspring of N-females was lower than those of D-females from artificial incubation, the maternal incubation do not cause social deprivation. Since D-females are survivors from artificial incubation, their offspring may also have had "good genes" to survive under artificial incubation. This may be a "positive" effect for ornamental fish producers, but it is a "negative" effect due to the alteration of maternal incubation for the *Tropheus* species itself.

Social deprivation can significantly negatively affect success rate of reproduction of species that are extinct in the wild or close to extinction and these species’ culture and rearing should be prioritised and well-managed in ornamental aquaculture to maintain them for the future. For this reason, and with respect to welfare principles, we strongly recommend similarly designed experiments focused on other potentially sensitive fish taxa. Once the syndrome is confirmed, we recommend avoiding artificial incubation of mouthbrooding fish in general.

## Methods

All methods, experiments, and experimental protocols were performed in accordance with the rules of the Czech University of Life Sciences Prague, Czech Republic, and law no. 246/1992 (The Prevention of Cruelty to Animals) and were approved by the Expert commission ensuring welfare of experimental animals at the mentioned university. The study was reported in accordance with ARRIVE guidelines (https://arriveguidelines.org).

### Model organisms

*Tropheus* sp. “Caramba” was chosen as model organisms to determine the effect of social deprivation syndrome in mouthbrooder fish due to their well-developed maternal mouthbrooding. The native range is at the eastern shore of the Ubwari Penninsula between Cape Caramba and Muzimu, Lake Tanganyika, Africa^35,48^, and this taxon is also referred to as *T*. sp. “black”^17,49^. All fish individuals used in the experiment were adults at least 2 years old. The groups were formed when the standard body size of females was approximately 8–10 cm, and 10–12 cm in males.

### Stocking

Parental shoals were obtained from the pet trade company Ingo-Pet. All individuals were collected in the wild in Lake Tanganyika and subsequently imported into the Czech Republic in 1991. Experimental groups were formed from offspring of this shoal and ongoing generations with respect to the optimal survival rate of these fish (15 males and 15 females in each group). All experimental individuals were of the same age and similar size. Within the experimental period, the females increased their standard body size by about 1–2 cm. When an individual or individuals died during the experiment, the entire group was removed from the analysis.

The diet content and frequency of feeding were the same in all tanks: commercially used food flakes (Tetra®, Germany), live and frozen plankton (*Artemia* sp., *Cyclops* sp., and *Daphnia* sp.) five times per day ad libitum. The temperature was 25–27 °C, pH 7.3–7.6, conductivity 450–550 μS/cm and light–dark cycle of 12:12 h. Each tank had a volume of 300 l and was equipped with a heater (Eheim, Germany), inner electric filters (Eheim, Germany), automatic water exchange, flat stones as spawning substrate, and sand on the bottom. The amounts of foods and water change frequency (50 L per day changed automatically) were not different among tanks.

### Maternally incubated individuals (N-individuals)

After spawning, females (mothers) from the parental shoal were kept with eggs and embryos in the pharyngeal cavity for 25–30 days with the co-specific shoal in the 300 l tanks as described above. From the beginning of the fourth week of the incubation period, mothers were kept individually in nurseries, where the fry was released approximately ten days later. As a nursery, a 62.5 l tank was used, furnished with a heater, an inner electric filter and a hideout for the mother (an empty flower-pot). The family was kept here for a further three to five days, till the fry was independent of mother’s care. The female was then removed from the nursery and released back into the one-species 300 l aquarium described above. Young individuals were reared in the nursery tank and after sex recognition (at the age of approximately 6 months), groups of thirty individuals of the same age were formed and stocked in 300 l experimental tanks. All these fish (including the wild-collected) were so-called “normal” (from the incubation perspective; hereafter labelled as N). In total, eight N-groups were formed.

### Socially deprived individuals (D-individuals)

Eggs in the eye spot stage, taken from the mouth of selected N-mothers kept in a parental shoal, were stocked in an incubator, consisting of laboratory glass cups of 150 ml volume, with water inflow from the inner electric filter, standing in a nursery tank. Water inflow was adjusted to ensure a moderate circulation of the incubated eggs and embryos. Free-swimming embryos and juvenile individuals were reared in 62.5 l tanks as described above. As in the N-individuals, after sex recognition (at the age of approximately 6 months), groups of thirty individuals of the same age and species (or colouration form) were formed and stocked in a 300 l experimental tank. The separated fish were considered “deprived” (from the incubation perspective; hereafter labelled as D). In total, fifteen D-groups were formed.

### Data collection

The reproduction in all experimental groups of both N and D-individuals was recorded from the second to fourth year of life of the members of each group within the period from 1994 to 2000 to obtain the number of maternally incubated hatchlings for comparison between both mentioned group types. At the beginning of the experiment, mean body size of the N and D-females was virtually the same, 86.1 mm and 87.3 mm, respectively (Mann–Whitney: nN = 15, nD = 15, z = − 0.560, P = 0.5755). The growth during the experiment had no effect on this relationship (final body size: N-females = 99.6 mm and D-females = 103.3 mm, z = − 1.452, P = 0.1466; Fig. S1). The number of laid eggs was counted in randomly selected spwanings in both groups (52 spawnings in N-females and 30 spawnings in D-females). After ritualised dancing, female laid usually one, rarely two or three eggs on upper side of sloped flat stone; the rolling egg was then captured by the female and deposited in its buccal cavity. Since the eggs are relatively big, this repeating process was prominent and total number of eggs laid during the each selected spawning within 10–15 min was recorded without disturbing the female. The spawning activity in the model species occurs just in the light period and female with a clutch of eggs in its buccal cavity is easily recognizable later, thus all the spawnings within the experiment were recorded.

Since females learn parental care progressively, the groups were observed for a period of 2 years, i.e. from the end of the second year till the end of the fourth year of life of individual experimental fish. Females were visually identified by body colouration patterns, body shape and size; the most similar individuals were marked by cutting off a small part of the upper or lower lobe of the caudal fin. A female carrying eggs in its buccal cavity was immediately identified by an enlarged pharyngeal pouch, reduced volume of the abdominal cavity, limited activity and lack of interest in food.

### Data analysis

To compare the number of spawnings by N and D-mothers, we employed a marginal model accounting for potential dependence of data collected within a particular breeding group^50^. We set deprivation (N versus D) as a fixed factor, breeding group as id, (quasi-) poisson distribution, log link function and independence correlation structure. Then, we ran a geeglm function as implemented in geepack (Generalized Estimating Equation Package)^51^.

The same approach we employed to compare the number offspring per spawning successfully reared by N and D-mothers. In this case, we set the model to account for mother identity instead of breeding group. We also included the egg laying order and its interaction in the initial full model. These fixed factors appeared not significant and were removed from the final reduced model (comparison between the full and the reduced models: df = 2, χ^2^ = 2.50, P = 0.290). The decision to control for mother identity was substantiated by a decomposition of variance in the square-root transformed number of reared offspring (performed in nlme package). The analysis revealed that 4.2% of the total variation is attributable to mother identity, while with breeding group less than 0.1%.

To compare the number of eggs laid by N and D mothers and their rearing success when artificially incubated separately from mother, we ran glms for poisson (log link function) and quasibinomial (logit link function) distribution, respectively. We present significance of the factor Deprivation from the analysis of deviance as well as parameters of the models. The calculations were performed in R-environment (R Core Team, 2021).

## Supplementary Information

Supplementary Information.

## Data Availability

The datasets used and analysed during the current study available from the corresponding author on reasonable request.
