# Supplementary material for: Social deprivation in maternal mouthbrooders Tropheus sp. “Caramba” (Teleostei: Cichlidae) decreases the success rate of reproduction and survival rate of fish fry
Source: Sci Rep. 2023 May 22;13:8284. doi: 10.1038/s41598-023-35467-z (PMC10203270; doi:10.1038/s41598-023-35467-z)
Supplement: Supplementary file 1 — Supplementary Information. [file 41598_2023_35467_MOESM1_ESM.docx]

**Supplementary material**

|  |  | **Reared offspring per spawning** | | |
| --- | --- | --- | --- | --- |
| **Group type** | **Spawnings** | **Median** | **Range** | **SD** |
| N | 53 | 6 | 0-16 | 3.8 |
| N | 52 | 7 | 0-12 | 3.1 |
| N | 55 | 7 | 0-14 | 3.3 |
| N | 58 | 6 | 0-16 | 3.7 |
| D | 29 | 0 | 0-3 | 0.6 |
| D | 28 | 0 | 0-3 | 0.6 |
| D | 34 | 0 | 0-2 | 0.6 |
| D | 29 | 0 | 0-3 | 0.6 |

**Tab S1:** Comparison of reproduction rate of maternally incubated and socially deprived females of *Tropheus* sp. “Caramba”: Group type (N = “normal” = maternally incubated; D = “deprived” = incubated separately from mother in artificial incubator); Spawnings = total number of spawnings of all females in each group within the period of two years; numbers of offspring per spawning successfully reared by mothers: median, range and standard deviation (SD).


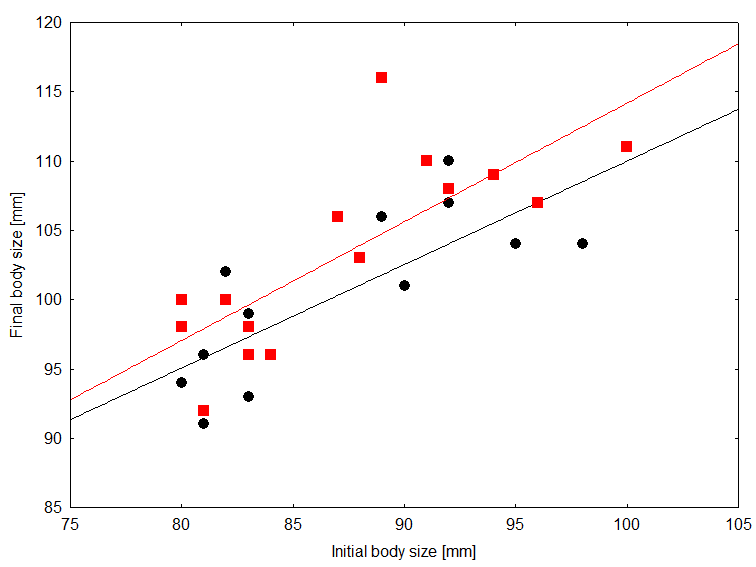


**Fig. S1:** Relationship between initial and final body size in selected groups of 15 N-females and 15 D-females during the experiment. Legend: black circles = N-females, red squares = D-females.
